# Clinical and pathological characteristics of IgG4-related interstitial lung disease

**DOI:** 10.3892/etm.2021.11018

**Published:** 2021-11-30

**Authors:** Xiaoting Lv, Feng Gao, Qicai Liu, Sheng Zhang, Zhihua Huang, Yongping Zhu, Haiyang Zong, Quwen Li, Sanyan Li

Exp Ther Med 15:1465–1473, 2018; DOI: 10.3892/etm.2017.5554

After the publication of the above article, the authors have realized that they made some inadvertent errors in the compilation of Figs. 2 and 3 during the preparation of their paper. Owing to the large number of clinicopathological images used in this paper, the author failed to classify them carefully, resulting in the misuse of a pair of the clinicopathological images specifically relating to Fig. 2C and Fig 3D.

The revised versions of Figs. 2 and 3, showing more representative data for Fig. 2C (the IgG4 immunohistochemical staining experiment that revealed numerous immunoreactive plasma cells) and for Fig. 3D (the immunofluorescence experiment that revealed obliterative phlebitis surrounded by lymphoplasmacytic infiltrates at the periphery of the field) are shown on the next page. Note that the revised data shown for these Figures do not affect the overall conclusions reported in the paper. The authors are grateful to the Editor of *Experimental and Therapeutic Medicine* for allowing them the opportunity to publish this corrigendum, and apologize for any inconvenience caused.

## Figures and Tables

**Figure 2:**
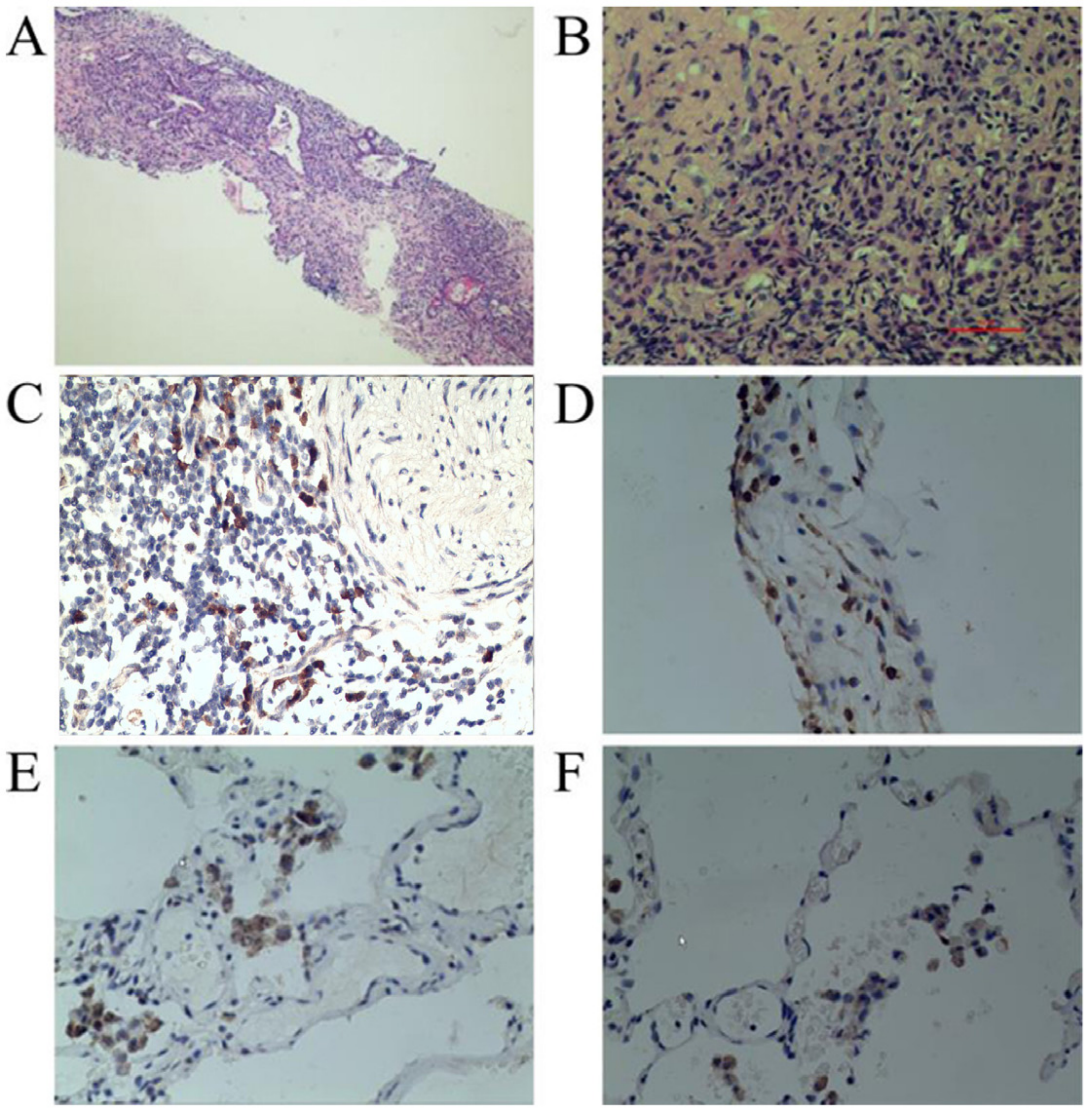
Histological characteristics of IgG4-related lung disease in case 1. (A) Solid nodular lesions stained with H&E had a mixed inflammatory infiltrate and fibrosis. Magnification, x100. (B) Inflammatory cells stained with H&E mainly consisted of lymphocytes and plasma cells. Some eosinophils were also observed and the number of alveolar epithelial cells was markedly reduced. Magnification, x400. (C) IgG4 immunohistochemical staining revealed numerous immunoreactive plasma cells. Magnification, x400. (D) Immunohistochemical analysis of the expressions of CD3 in serial sections of IgG4-related lung disease. Magnification, x400. (E) Immunohistochemical analysis of the expression of CD20 in serial sections of IgG4-related lung disease. Magnification, x400. (F) Immunohistochemical analysis of the expressions of CD38 in serial sections of IgG4-related lung disease. Magnification, x400. H&E, hematoxylin and eosin; IgG4, immunoglobulin G4; CD, cluster of differentiation.

**Figure 3:**
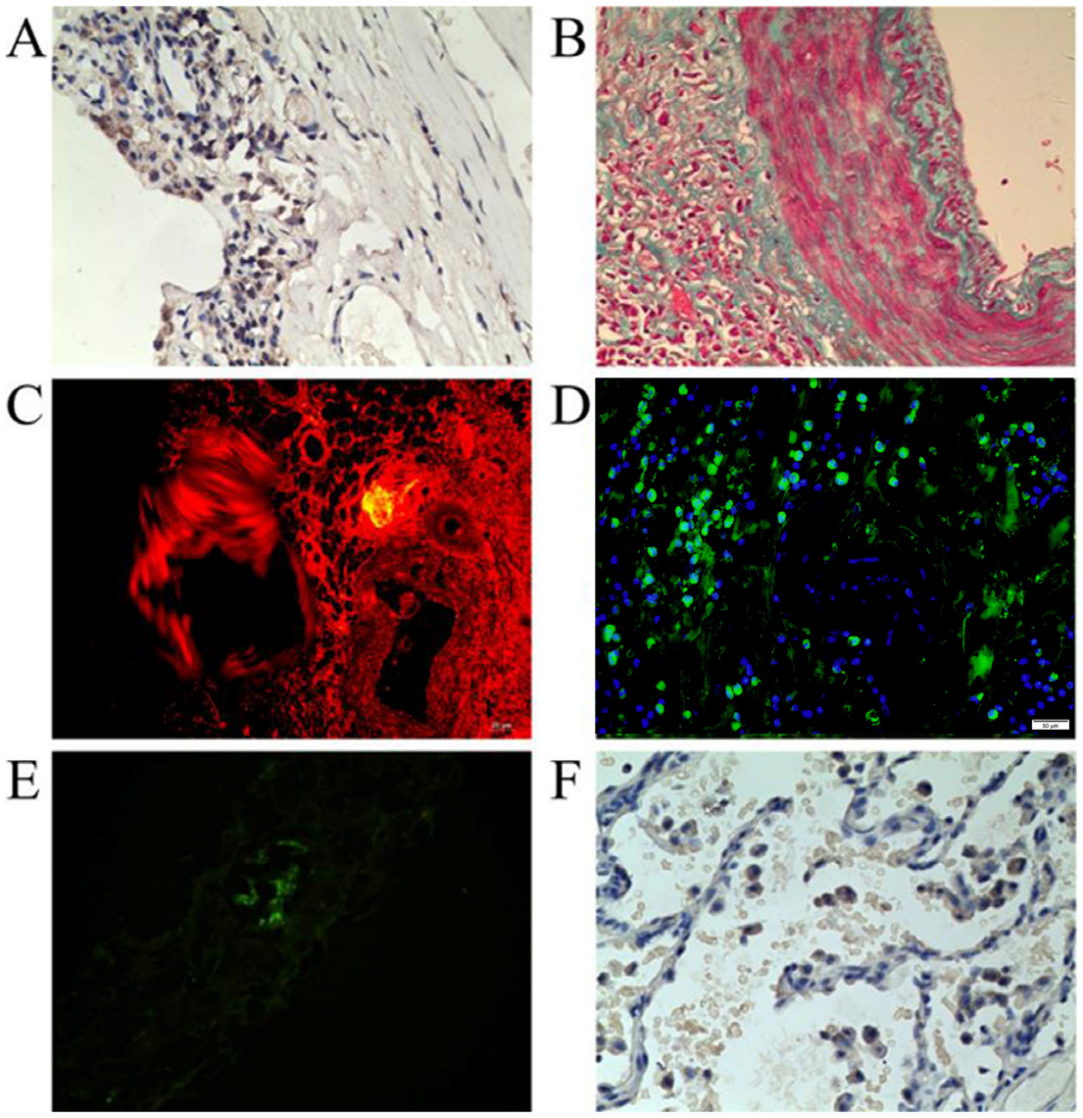
Immunohistochemical analysis of the expression of (A) CD68 in serial sections of IgG4-related lung disease. Magnification, x400. (B) Elastica-van Gieson staining showed collagen fiber diffuses hyperplasia on the vessel wall, followed with collagen aggradation surrounding the vessels. Magnification, x200.(C) Storiform fibrosis was observed using immunofluorescence staining. Magnification, x400. (D) Immunofluorescence revealed obliterative phlebitis surrounded by lymphoplasmacytic infiltrates at the periphery of the field. Magnification, x400. (E) IgG4 antibody fluorescence of lung tissue surrounding the blood vessels. Magnification, x200. (F) Immunohistochemical analysis of α-1-AT in IgG4-related lung disease. All samples are from case 1. α-1-aAT, α-1-antitrypsin.

